# Mouse Spexin: (I) NMR Solution Structure, Docking Models for Receptor Binding, and Histological Expression at Tissue Level

**DOI:** 10.3389/fendo.2021.681646

**Published:** 2021-07-02

**Authors:** Matthew K. H. Wong, Mulan He, Kong Hung Sze, Tao Huang, Wendy K. W. Ko, Zhao-Xiang Bian, Anderson O. L. Wong

**Affiliations:** ^1^ School of Biological Sciences, The University of Hong Kong, Hong Kong, Hong Kong; ^2^ Department of Microbiology, Queen Mary Hospital, University of Hong Kong, Hong Kong, Hong Kong; ^3^ School of Chinese Medicine, Hong Kong Baptist University, Hong Kong, Hong Kong

**Keywords:** GalR2/3, nuclear magnetic resonance structure, receptor docking, tissue expression, mouse, spexin

## Abstract

Spexin (SPX), a highly conserved neuropeptide, is known to have diverse functions and has been implicated/associated with pathological conditions, including obesity, diabetes, anorexia nervosa, and anxiety/mood disorders. Although most of the studies on SPX involved the mouse model, the solution structure of mouse SPX, structural aspects for SPX binding with its receptors GalR2/3, and its cellular expression/distribution in mouse tissues are largely unknown. Using CD and NMR spectroscopies, the solution structure of mouse SPX was shown to be in the form of a helical peptide with a random coil from Asn^1^ to Pro^4^ in the N-terminal followed by an α-helix from Gln^5^ to Gln^14^ in the C-terminus. The molecular surface of mouse SPX is largely hydrophobic with Lys^11^ as the only charged residue in the α-helix. Based on the NMR structure obtained, docking models of SPX binding with mouse GalR2 and GalR3 were constructed by homology modeling and MD simulation. The models deduced reveal that the amino acids in SPX, especially Asn^1^, Leu^8^, and Leu^10^, could interact with specific residues in ECL_1&2_ and TMD_2&7_ of GalR2 and GalR3 by H-bonding/hydrophobic interactions, which provides the structural evidence to support the idea that the two receptors can act as the cognate receptors for SPX. For tissue distribution of SPX, RT-PCR based on 28 tissues/organs harvested from the mouse demonstrated that SPX was ubiquitously expressed at the tissue level with notable signals detected in the brain, GI tract, liver, gonad, and adrenal gland. Using immunohistochemical staining, protein signals of SPX could be located in the liver, pancreas, white adipose tissue, muscle, stomach, kidney, spleen, gonad, adrenal, and hypothalamo-pituitary axis in a cell type-specific manner. Our results, as a whole, not only can provide the structural information for ligand/receptor interaction for SPX but also establish the anatomical basis for our on-going studies to examine the physiological functions of SPX in the mouse model.

## Introduction

Spexin (SPX), also called neuropeptide Q, is a recent example of identifying novel peptides using bioinformatic approach prior to their purification/functional studies (1, 2). In human, SPX is encoded by the c12orf39 gene located in chromosome 12 (3) and confirmed to be a secreted peptide by functional expression in cell lines (e.g., β-TC3/COS-7 cells) (1, 3), probably after protein processing in Golgi and endoplasmic reticulum (3). The mature peptide of SPX is highly conserved from fish to mammals (4, 5), and recent phylogenetic analysis and comparative synteny reveal that SPX is co-evolved with galanin as a result of whole genome duplication occurred during vertebrate evolution (4, 6). Functional studies also confirm that the galanin receptors GalR2 and GalR3 but not GalR1 can act as the cognate receptors for SPX (6). In animal models, and to a less extent in human studies, SPX was found to be widely expressed at tissue level (7, 8) and involved in diverse functions, including stomach contraction (1), GI tract movement (9), energy balance and weight loss (10), bile acid synthesis (11), appetite control (12, 13), glucose homeostasis (14, 15), lipid metabolism (16, 17), reproduction (18, 19), pain perception (20, 21), stress/anxiety (22, 23), and cardiovascular/renal functions (24). Besides its proposed functions as a neurotransmitter/neuromodulator, which is consistent with high levels of SPX expression in different brain areas (4), increasing evidence also support the emerging role of SPX as a neuroendocrine factor (5), e.g., for regulation of gonadotropins (18, 19) and adrenal steroids (25). In human subjects, notable changes in SPX expression/serum level can be associated with pathological conditions/diseases, e.g., in type I/II (7, 26) or gestational diabetes (27), childhood (28, 29) and adult obesity (30), metabolic syndrome (31), cardiovascular disease (32), and anorexia nervosa/other psychiatric disorders (33, 34), which have aroused the interest of using SPX as a new target for drug design with clinical implications (22, 35).

In previous reports on elucidating the functions of SPX, e.g., in gut motility (9), fatty acid uptake (10, 16), locomotor activity and lipid metabolism (10), nociception (20), and antidepressive/anxiolytic activity (22), the mouse has been used as a major model for both *in vivo* and *in vitro* studies. However, the solution structure of mouse SPX, structural aspects for SPX interaction with mouse GalR2/3, and cellular expression of SPX in various tissues of the mouse, especially those with endocrine functions, are largely unknown. Regarding the functional role of SPX in feeding regulation, our study in goldfish has shown that SPX can serve as a satiety factor inducible by food intake and the postprandial rise in SPX can inhibit feeding behavior *via* differential regulation of orexigenic (e.g., NPY, AgRP, and Apelin) and anorexigenic signals (e.g., POMC, CART, and CCK) expressed in brain areas involved in appetite control (12). After feeding, the rise in insulin caused by glucose uptake can serve as a functional link between food intake and SPX expression and the SPX responses induced by insulin are mediated by PI3K/Akt and P_38_^MAPK^ cascades coupled to activation of insulin receptor and IGF-I receptor (36). Whether similar mechanisms also occur in mammals and contribute to the weight loss reported after SPX treatment in the mouse (10) are still unclear. To shed light on the comparative aspects of SPX in feeding control, our study has been extended to the mouse model. Our findings are presented in this three-paper series with focus on (i) defining the structural features for SPX : GalR2/3 interaction and mapping the target sites of expression for SPX, (ii) elucidating the mechanisms for feeding regulation by SPX in terms of the postprandial responses of SPX at tissue level and the effects of SPX on food intake and central expression of feeding regulators, and (iii) unveiling the role of glucose and insulin in regulating SPX expression and its functional implications in feeding control in the mouse. In part I of this three-paper series, circular dichroism (CD) and nuclear magnetic resonance (NMR) spectroscopies were used to set up the solution structure of mouse SPX, which was then subjected to molecular dynamics (MD) simulation to deduce the docking models for SPX binding with mouse GalR2 and GalR3, respectively. To map the tissues/organs with SPX expression, which can serve as the target sites for the postprandial responses of SPX, RT-PCR coupled to immunohistochemical staining (IHS) were used to examine the cellular expression of SPX in tissues related to endocrine functions and/or with biological actions reported for SPX. Our studies not only can provide new information on the structural aspects for SPX binding with GalR2/3 but also lay down the anatomical basis for our parallel study on SPX expression induced by food intake in the mouse model.

## Materials and Methods

### Animals and Tissue Sampling

Mice (*Mus musculus*) of the C57BL/6N strain with body weight of 25 to 35 g were housed by group caging (four animals per cage) at 22°C and 60% relative humidity under a 12-h dark:12-h light photo period and fed *ad libitum* with standard chow for rodents. On the day of tissue sampling, the mice were anesthetized by co-treatment with xylazine (10 mg/kg) and ketamine (80 mg/kg). After that, the animals used for RT-PCR were decapitated, and target tissues/organs were rapidly excised, frozen in liquid nitrogen, and homogenized in TRIZOL (Invitrogen, Carlsbad, CA) for RNA extraction using a Tissue Lyser LT (Qiagen, Valencia, CA). For sample preparation for IHS, the animals under anesthesia were transcardially perfused with ice-cold PBS (pH 7.4, with 10 U/ml herparin) followed by Zamboni fixative at 1.5 ml/min using a peristalic pump. After that, target tissues/organs were excised and post-fixed in Zamboni fixative at 4°C for 15 to 18 h followed by standard procedures for ethanol dehydration and paraffin embedding. Tissue sampling was conducted according to the protocol 3878-16 approved by the Committee on the Use of Live Animal in Teaching and Research at the University of Hong Kong (Hong Kong).

### Solution Structure of Mouse SPX Deduced by CD and NMR Spectroscopies

The 14 a.a. mature peptide of mouse SPX (NWTPQAMLYLKGAQ, 98.2% pure) was synthesized by GenScript (Piscataway, NJ) and used for CD analysis with J-720 spectropolarimeter (Jasco, Tokyo, Japan) in a solvent system composed of distilled water and trifluoroethanol (TFE) and with increasing levels of TFE from 0% to 100%. The CD spectra of SPX were monitored at 0.2 nm steps from 190 to 250 nm using a scanning speed of 50 nm/min with the solvent containing the corresponding levels of TFE as the background control. The levels of secondary structure elements detected in SPX with different solvent backgrounds were deduced from the CD data by deconvolution analysis using CDPRO software (http://lamar.colostate.edu/~sreeram/CDPro/main.html). Using solvent system with optimized level of TFE, NMR experiments, including DQF-COSY, TOCSY, and NOESY. were conducted for SPX with Avance 600 NMR spectrometer (Bruker Biospin, Rheinstetten, Germany) as described previously (12). DQF-COSY and TOCSY were used to identify the spin systems in SPX molecule, and NOESY was used to establish the sequential connectivity between the cross-peaks. Data processing and cross-peak assignment were conducted using Topspin and Sparky 3.110 (http://www/cgl.ucsf.edu/home/sparky) and computation of SPX structure was carried out using Cyana 2.1 (http://www.cyana.org/). Data for NOE intensity and chemical shift extracted by Sparky 3.110 were used for structural calculation with Cyana 2.1. After multiple cycles of iterative structural calculation, the data for upper limit distance constraints were manually checked and used in extensive simulated annealing protocol with 10,000 steps to generate >100 final refined structures for SPX. Among these structures, structural evaluation and statistical analysis were performed in the 20 energy-minimized conformers with the top scores in potential energy function and the data were then used to construct the 3D model and surface plot of SPX with MOLMOL software (http://jedi.mathstat.dal.cal/MolMOL/).

### SPX Docking With GalR2/3 by Homology Modeling and MD Simulation

The 3D models for mouse GalR2 and GalR3 were constructed by homology modeling as described previously (9). Briefly, the protein sequences of mouse GalR2 (Uniprot ID O88854) and GalR3 (Uni-prot ID O88853) were submitted to Protein Data Bank to pull out the appropriate template with crystal structure. Based on BLAST search with a low complexity mask, the crystal structure 4EA3 (with 3.0 Å resolution) for human OPRL1 receptor binding with a C-24 peptide mimetic (37) was selected for GalR2/3 modeling as (i) OPRL1 and GalR2/3 can be grouped in the same clade in phylogenetic analysis of GPCR evolution, (ii) OPRL1 shares a high degree of a.a. sequence homology with GalR2/3 (68.4% for GalR2 and 75.1% for GalR2), and (iii) the bound ligand for 4EA3 is a peptide mimetic supposed to have binding property similar to small peptides including SPX. The 3D models of GalR2/3 were constructed within a lipid bilayer simulation based on the pdb data of 4EA3 by Protein Builder Module for homology modeling in MOE server (https://www.chemcomp.com/MOE-Molecular_Operating_Environment). Assignment of ionization states and hydrogen coordinates were conducted by Protonate 3D of MOE tool pack and 50 intermediate models were generated during the process of main chain and side chain sampling. Medium refinement was performed for both the intermediate and final models with energy minimization using Amber12:EHT forcefield and R-field solvation model. The final models for GalR2/3 were selected based on the level of structural compactness and free energy scores for hydration and electrostatic solvation using the Generalized Bom/Volume Integral (GB/VI) model.

After aligning the final models for GalR2/3 using the respective first principal axis and introducing heterogeneous lipids into the membrane bilayer (POPC in upper leaflet and POPE in lower leaflet) and KCl (0.15M) to neutralize the charge of the system with Monte-Carlo placing method, MD simulation of SPX binding with GalR2/3 was conducted using LEaP module of AMBER 18 (http://ambermd.org/GetAmber.php) with ff12SB : Lipid14 forcefield and the structural data for NMR solution structure of mouse SPX following the Schrodinger’s Induced-Fit protocol (https://www.schrodinger.com/induced-fit). Flexible docking of SPX was carried out by the proxy triangle placement method in the simulated ligand:receptor complex solvated in a triclinical periodic box under the TIP3P water model and proper neutralization by counter-ions with an isothermal-isobaric NPT ensemble. During the process, a 10-ns production simulation was performed with cluster analysis of the associated trajectories, and hydrogen-mediated interactions were constrained by SHAKE method using a 2-fs integration time step, whereas the non-bonded interactions involving long-range electrostatics/Van der Waals force were handled by PME method. The docking poses obtained were screened to exclude those with SPX binding outside the binding pocket in GalR2/3, and the remaining poses were further refined by molecular mechanics with GBSA calculations for interacting residues between the ligand and receptor within a distance of 5 Å. At least 50 poses were retained for each GalR subtypes during the placement and refinement steps, and the poses with the top scores based on GBVI/WSA dG method were used for the deduction of binding free energy and ligand potential energy followed by contact analysis for potential interactions of the residues between SPX and GalR2/3 in the final docking models.

### Tissue Distribution of SPX Expression by RT-PCR and IHS

Tissue expression profile of SPX was established in mouse using RT-PCR. Briefly, total RNA was isolated from 28 selected tissues/organs with TRIZOL, digested with DNase I to remove genomic DNA contamination, and reversely transcribed using Superscript II (Invitrogen). RT samples obtained were then used as the templates for real-time PCR using primers specific for mouse SPX (forward primer: TGACACAAGTGAGTGCCACA; reverse primer: ACACACACACACACACACGG) with a QuantiTect SYBR Green RT-PCR kit (Qiagen, Hilden, Germany). Real-time PCR was conducted in a Rotor Gene-Q qPCR System (Qiagen) for 35 cycles with denaturation at 94°C for 30 s, annealing at 65°C for 30 s, and extension at 72°C for 30 s. By the end of each PCR cycle, SYBR green signals were captured at 82°C for 20 s. The authenticity of PCR product (165 bp in size with *Tm* at 87°C) was routinely confirmed by melting curve analysis after the assay. Serial dilutions of plasmid DNA with the sequence of mouse SPX were used as the standards for data calibration, and parallel measurement of GAPDH mRNA was used as the internal control.

For cellular expression of SPX signals, IHS for SPX immunoreactivity was performed in sections (10 μm in thickness) prepared from selected tissues/organs after fixing in Zamboni fixative using a rabbit antiserum for mammalian SPX (H-023-81, Phoenix Pharmaceuticals, Burlingame, CA). For validation of antiserum specificity, displacement studies were conducted using a SPX ELISA kit with the same antiserum (EK-02-81, Phoenix Pharmaceuticals) in the presence of increasing levels of fish/mouse SPX (amidated and non-amidated), galanin/kisspeptin (peptides co-evolved with SPX in the same lineage), and unrelated peptides within similar size (e.g., NPY, VIP, PACAP, αMSH, secretin, and orexin). After confirming the specificity of SPX antiserum, IHS was conducted based on the standard protocol in our laboratory (38). Briefly, sections were blocked with normal goat serum (2.5%) and incubated with SPX antiserum (1:600) for 15 to 18 h at 4°C. Parallel treatment with the same dilution of normal rabbit serum (NRS) was used as a negative control. After incubation with SPX antiserum, the sections were treated with biotin-conjugated goat anti-rabbit antibody (1:400, Vector Lab, Burlingame, CA) followed by signal development using the avidin-biotin complex reagent (Vector Labs) and avidin-conjugated HRP using diaminobenzidine (Sigma-Aldrich, St. Louis, MO) as the substrate. Unless stated otherwise, the sections were counterstained with hematoxylin followed by permanent mounting with DPX mountant (Sigma-Aldrich). Because sexual dimorphism of SPX expression was not apparent at the tissue level, the results for IHS and RT-PCR from the male and female animals (except for the testes and ovary) were pooled together for data presentation.

### Data Transformation and Statistical Analysis

For real-time PCR of SPX/GAPDH mRNA levels, standard curves constructed with serial dilutions of plasmid DNA carrying the ORF/amplicon of the respective targets with a dynamic range of ≥ 10^5^, amplification efficiency ≥ 0.98, and correlation coefficient of ≥ 0.95 were used for data calibration with the RotorGene Q-Rex software (Qiagen). To control for different amount of tissue used in RNA extraction, the raw data for qPCR (in femtomole target transcript detected) were normalized as a ratio of genomic DNA (in µg) harvested in the same sample during RNA isolation by TRIZOL according to the instructions by the vendor. Normalized data for SPX and GAPDH in different tissues, expressed as mean ± SEM (N = 5), were subjected to Student’s t-test with the corresponding gene expression in the skin (the tissue with the lowest level of SPX signal) as a reference control. Differences were considered as significant when the *p* value for comparison was below 0.05 level.

## Results

### Solution Structure of Mouse SPX Deduced by CD and NMR Spectroscopies

**Figure 1:**
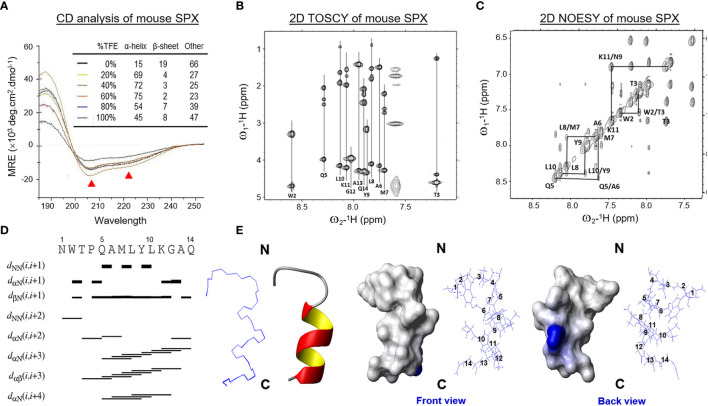
Solution structure of mouse SPX. **(A)** Analysis of secondary structure elements in mouse SPX by CD spectroscopy. The CD spectra of mouse SPX (NWTPQAMLYLKGAQ) were obtained in a solvent system composed of distilled water with increasing levels of TFE (0–100%) and the relative contents of secondary structure elements were deduced by deconvolution analysis based on the readings of mean residue ellipticity (MRE). (Ellipticity minima at 208 and 222 nm indicative of helical structures were marked by red triangles.) Identification of spin systems for individual a.a. in mouse SPX and their through-bond/through-space correlations by TOCSY and NOESY experiments conducted in 60% d4-TFE at 298K. Data presented are the fingerprint region showing C_α_-N_α_H ^1^H-NMR cross-peaks of 2D TOSCY **(B)** and NH-NH region with N_α_H_i_-N_α_H_i+1_^1^H-NMR cross-peaks of 2D NOSEY of mouse SPX **(C)**. **(D)** Delineation of helical region in mouse SPX by NOE connectivities deduced from TOCSY and NOESY spectra. Sequential dNN connectivities and assignments indicate the presence of an α helical structure from Gln^5^ to Gln^14^ in mouse SPX. **(E)** Stereoview superposition of final restrained structures (in ribbon plot) and stereo model of mouse SPX (in surface plot). In the stereo model, both the front and back views are presented with basic a.a. in blue and neutral residues in white.

Since small peptides are known to exist in the form of random coil in solution and start to fold into proper conformation for receptor binding in close proximity to the plasma membrane of target cells, a solvent system with proper hydrophobicity, presumably mimicking the microenvironment close to the cell surface, would be required for the NMR studies for mouse SPX. To validate the solvent system for NMR experiments, the mature peptide of mouse SPX was dissolved in a TFE: H_2_O solvent system with increasing hydrophobicity by gradually elevating the ratio of the organic co-solvent TFE (0–100%) and subjected to far-UV CD spectroscopy. As shown in **Figure 1A**, the CD spectra with the negative ellipticity minima at 208 and 222 nm (indicative of α helical structures) became quite distinct with 60% TFE. Deconvolution analysis revealed that 60% TFE could allow for the maximal establishment of helical component (75%), which is equivalent to ~10 a.a. residues in SPX molecule involved in the formation of α helical structure. Given that the levels of β sheet structure deduced after adding TFE were found to be rather low (2–8%), it would be logical to assume that β sheet does not form a major component in the stable conformation of SPX under a hydrophobic environment.

Using the optimized solvent system (60% TFE), 2D TOCSY and DQF-COSY experiments (for through-bond correlations) were conducted using ^1^H-NMR spectroscopies to define the spin systems for individual a.a. residues within the SPX molecule and deduce the side chain connectivities through the cross-peaks to high field (**Figure 1B**). Sequence-specific assignment of the spin systems related to *d_NN_* connectivities was performed by 2D NOESY (for through-space correlation, **Figure 1C**). Based on the data for NOE assignment and chemical shift obtained (**Table 1**), secondary structure elements in SPX were mapped using NOE connectivities and ^1^H_α_ chemical shift index (**Figure 1D**) and the pattern of the medium-range NOEs for N_α_H*_i_* and N_α_H*_i_*_+3_ residues indicates that the mouse SPX has an extended α helix covering Gln^5^ to Gln^14^ in the C-terminal. However, the cross-peak signals for N_α_H*_i_* and N_α_H*_i_*_+1_ residues were weak/undetectable for the region covering Asn^1^ to Pro^4^, implying that the N-terminal of mouse SPX does not have detectable level of secondary structure elements.

**Table 1:** ^1^H assignment and chemical shift data for mouse SPX (60% TFE at 298 K).

Spexin	NH	αH	βH	γH	δH	Others
Asn 1	6.952					
Trp 2	7.993	4.091	2.715, 2.661			
Thr 3	6.601	3.999	3.784	0.650		
Pro 4		3.540	1.726, 1.313	1.464, 1.396	3.135, 2.919	
Gln 5	7.683	3.370	1.443	1.891, 1.755		
Ala 6	7.144	3.544	0.886			
Met 7	7.096	3.699	1.602	2.118, 2.008		
Leu 8	7.222	3.495	1.197, 1.131	0.924	0.310, 0.267	
Tyr 9	7.273	3.724	2.588, 2.530			H-2/6, 6.510; H-3/5, 6.215
Leu 10	7.528	3.540	1.329, 1.287	1.030	0.388	
Lys 11	7.465	3.596	1.369	0.974, 0.919	1.124	EH, 2.412; ζNH_3_^+^, 6.992
Gly 12	7.421	3.342				
Ala 13	7.343	3.668	0.808			
Gln 14	7.300	3.691	1.872, 1.808	1.618, 1.480		

Based on NOE cross-peak data and proton-proton distance constraints revealed at the atomic level, 3D structure of SPX was deduced using MD calculations and the results of structural analysis and QC statistics were summarized in **Supplemental Table 1**. Briefly, 159 nonredundant upper-limit distance constraints, including 106 short-range, 49 medium-range, and 4 long-range distance constraints, were generated by CYANA 2.1 based on the cross-peak data by NOESY. The best 20 geometry structures with the lowest levels of target function (< 0.05 Å^2^), distance constraint violation (< 0.2 Å), and angle constraint violation (< 5°) were selected for further MD refinement to produce the final model of SPX solution structure (**Figure 1E**). During the process, the backbone traces of the final 20 energy-minimized structures were superimposed over the helical region and the root mean square derivation (RMSD) of these ensembles was found to be well below 0.1 Å with the backbone dihedral angles (Φ and Ψ) all within the most favored region according to Ramachandran analysis. As shown in the ribbon plot of the final ensembles of the 20 energy-minimized structures, the N-terminal of mouse SPX from Asn^1^ to Pro^4^ is in the form of a random coil followed by an α helix from Gln^5^ to Gln^14^ in the C-terminal (**Figure 1E**, left panels), which is consistent with the idea of ~10 a.a. involved in α helix formation predicted by CD analysis. The surface plot for the average structure of these final ensembles also reveals that the overall surface of mouse SPX is largely hydrophobic with Lys^11^ located in the helical region as the only charged residue on the molecular surface (**Figure 1E**, right panels).

### Homologous Modeling of Mouse GalR2/3 With SPX Docking by MD Simulation

**Figure 2:**
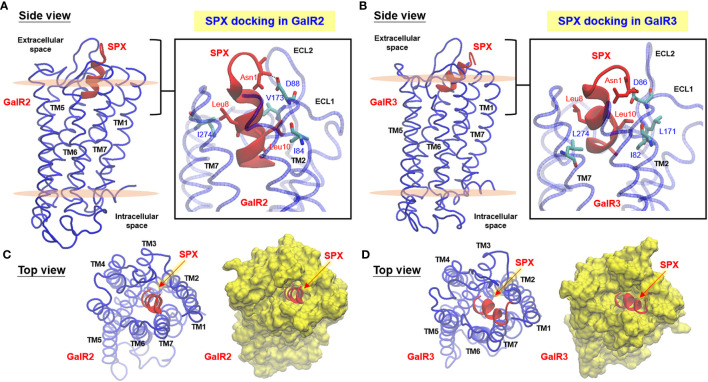
Docking models for SPX binding with mouse GalR2 and GalR3. Side view of homologous modeling of mouse GalR2 **(A)** and GalR2 **(B)** within an asymmetric POPC/POPE lipid bilayer with MD simulation of ligand binding by mouse SPX. Molecular interactions in the well-conserved contact sites in SPX (Asn^1^, Leu^8^, and Leu^10^) with the residues in GalR2 (Ile^84^, Asp^88^, Val^173^, and Ile^274^) and GalR3 (Ile^82^, Asp^86^, Leu^171^, and Leu^274^) are presented in the blow-up view close to the surface in the inset of **(A, B)**, respectively. Insertion of the helical region of SPX into the central binding pocket formed by clustering of TMD_1–7_ of GalR2 **(C)** and GalR3 **(D)** could also be noted in the top view of the respective docking models. The helical model of SPX deduced by NMR is marked in red and the corresponding structures for GalR2/3 (including TMD_1–7_, ECL_1–3_, and ICL_1–3_) are presented in blue for the ribbon plot or in yellow for the surface plot of the respective models. Meanwhile, the upper and lower surface of the lipid bilayer in the side view of the docking models are presented in pink color.

Since the TMD_1–7_ in class A GPCRs are highly conserved compared with other subclasses, the crystal structure of OPRL1, a nociceptin receptor belonging to class A GPCR and closely related to GalR1-3 based on phylogenetic analysis, was used as a template for homologous modeling of mouse GalR2/3. After sequence alignment of OPRL1 with GalR2/3 to remove the gaps in ECL_2/3_ and ICL_3_ followed by introduction of disulfide bond between the well-conserved Cys residues in TMD_3_ and ECL_2_ and “ionic lock” structure in DRY motif of TMD_3_, *in silico* modeling of GalR2/3 was conducted with simulation of the receptor embedding in an asymmetric POPC/POPE membrane bilayer (**Figures 2A, C** **)**. During the process, the N- and C-terminals of GalR2/3 were omitted for structural simulation as suggested by previous reports for human GalR1-3 modeling (39, 40), mainly due to (i) the short N-terminal of GalR1-3 has a high degree of sequence variations but with no notable secondary structures, and (ii) the C-terminal tail in general is not involved in the formation of binding pocket in GPCR. As shown in the energy-minimized models after optimization of backbone coordinates and placement of side chains, the seven α helixes of TMD_1–7_ in mouse GalR2 (**Figures 2A, C** **)** and GalR3 (**Figures 2B, D**) can be clustered into a “compact core” with a central binding pocket typical of GPCR by counter-clockwise arrangement of TMD_1–7_ when viewed from the extracellular side. In both cases, TMD_4_, TMD_6_, and TMD_7_ exhibit an almost perpendicular orientation related to the membrane surface whereas the other TMDs are tilted at ~30° along the vertical axis. Meanwhile, the ECL_2_ between TMD_4_ and TMD_5_, ICL_3_ between TMD_5_ and TMD_6_, and ECL_3_ between TMD_6_ and TMD_7_ are predicted to be highly flexible in these models. In accordance to the “positive-inside” rule for GPCR modeling (41), the number of positively charged residues identified at the boundary of α helixes and intracellular side (10–12 for GalR2/3) was shown to be notably higher than that of the other side facing extracellular space (2–3 for GalR2/3). Parallel Ramachandran plots for GalR2 and GalR3 (for model reasonableness) also revealed that most of the structural data for back-bone dihedral angles (> 90%) were within the most favored region and with a small fraction in the additional allowed region (**Supplemental Figure 1**). These results, as a whole, indicate that our models for the two mouse receptors are geometrically and energetically stable, and presumably, in a favorable state for ligand binding.

Given that the solution structure deduced by NMR is commonly accepted to be the conformation for receptor binding in small peptides, the NMR structural data of mouse SPX were used for induced-fit docking with MD simulation to study SPX binding with the newly established models for mouse GalR2 (**Figure 2A**) and GalR3 (**Figure 2B**), respectively. During the process, the docking poses derived from clustering analysis of the trajectories based on a 10-ns production simulation could be separated into a major cluster with the C-terminal α helix of SPX inserted into the binding pocket of GalR2/3 and a minor cluster with the N-terminal random coil of SPX buried in the same binding site. Our structural analysis was focused on the first docking mode as (i) the number of docking poses obtained for the first case was much higher than the latter, indicating a better representation for ligand:receptor interaction, and (ii) a recent study has reported that the N-terminal attachment of a large chemical group (e.g., Fmoc/PEG) does not modify the effects of SPX and its peptide analogs on GalR2/3 activation (42), suggesting that the N-terminal of SPX may not be essential for receptor binding. After structural refinement (with Cα RMSD ≤ 2 Å), the docking poses with the top scores for binding free energy (an index for ligand: receptor interaction energy) and ligand conformation energy (an index for strain energy of bound ligand) were used for contact analysis for SPX : GalR2/3 binding. In the final models obtained, the residues in SPX, including Asn^1^, Trp^2^, Gln^5^, Leu^10^, Lys^11^, Gly^12^, Ala^13^, and Gln^14^, were shown to interact selectively with specific residues in the ECL_1_, ECL_2_, TMD_2_, TMD_3_, and TMD_7_ of mouse GalR2 and ECL_1_, ECL_2_, ECL_3_, TMD_2_, and TMD_7_ of mouse GalR3 *via* hydrogen bonding, ionic interaction and/or hydrophobic contact (**Table 2**). Except for Asn^1^ and Trp^2^, which interact with ECL_1/2_, the other interacting residues identified in SPX are all within the helical region in the C-terminal and interact with the complementary residues in TMD_2_, TMD_3_, and TMD_7_ located in the binding pocket predicted by our GalR2/3 modeling. By comparing the docking complexes of SPX with GalR2 (**Figure 2A**, inset) and GalR3 (**Figure 2C**, inset), the interactions of Asn^1^ with ECL_1_ (via Asp^88^ in GalR2 and Asp^86^ in GalR2), Leu^8^ with TMD_7_ (via Ile^274^ in GalR2 and Leu^274^ in GalR3), and Leu^10^ with ECL_2_ (via Val^173^ in GalR2 and Leu^171^ in GalR3) and TMD_2_ (via Ile^84^ in GalR2 and Ile^82^ in GalR3) are well conserved between the two receptors, suggesting that these residues are key elements in SPX molecule for receptor binding. Of note, the binding free energy for the two receptors (−19.74 kcal/mol for GalR2 and −19.62 kcal/mol for GalR3) were found to be well above the level (−5 to −10 kcal/mol) required for high affinity binding (43) and the high levels of ligand conformation energy deduced for SPX (−363.69 kcal/mol for GalR2 and −356.85 kcal/mol for GalR3) are also consistent with our models with mouse SPX maintained/constrained in a helical configuration for ligand docking in the binding pocket of the respective receptors.

**Table 2:** Predicted interactions between SPX and GalR2/3.

Spexin	GALR2			GALR3		
	Residue^1^ / Position^2^		Interaction type^3^	Residue / Position		Interaction type
Asn1	**D88**	**ECL1**	**HB; ION**	**D86**	**ECL1**	**HB; ION**
Trp2	L168	ECL2	HC	D86	ECL1	HB
Gln5	S276	7.38	HB	S266	7.28	HB
Leu8	Y270	7.32	HB	F265	ECL3	HC
	**I274**	**7.36**	**HC**	**L274**	**7.36**	**HC**
Leu10	**I84**	**2.64**	**HC**	**I82**	**2.64**	**HC**
	**V173**	**ECL2**	**HC**	**L171**	**ECL2**	**HC**
Lys11	Q81	2.61	HB	Q79	2.61	HB
	Y85	2.65	HB			
Gly12	R267	7.29	HB	R262	ECL3	HB
Ala13	H101	3.29	HB			
	R267	7.29	HB			
Gln14	Y85	2.65	HB	C172.O	ECL2	HB
				R262.NH2	ECL3	HB

^1^Conserved interactions in SPX:GalR2/3 complex were bold-typed.

^2^Position of a.a. residues were numbered based on GPCRdb numbering scheme.

^3^HB, hydrogen bonding; ION, ion bonding; HC, hydrophobic contact.

### Tissue Expression of SPX Revealed by RT-PCR and IHS

**Figure 3:**
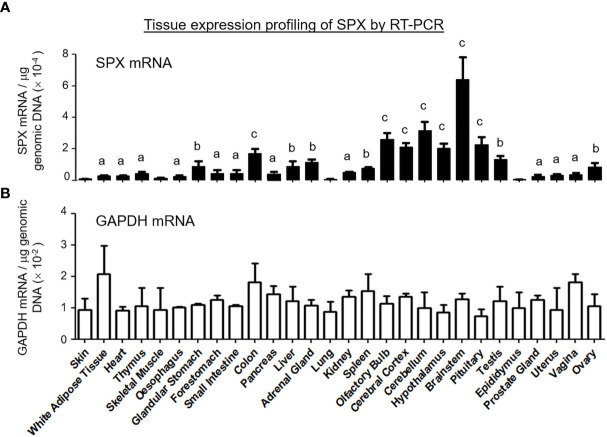
Tissue distribution of SPX expression by RT-PCR. A total of 28 tissues were harvested from the mouse, reversely transcribed and subjected to real-time PCR with primers specific for mouse SPX. **(A)** The data for SPX transcript were normalized with genomic DNA obtained in the same sample during the process of RNA isolation to adjust for variations in the amount of tissue used in sample preparation. Parallel PCR for GAPDH with similar data normalization was also conducted to serve as the internal control. **(B)** The groups denoted with different letters ^(a, b, c)^ represent a significant difference (*P* < 0.05, Student’s *t* test, N = 5) in SPX gene expression compared to the skin (the tissue with the lowest level of SPX expression as a reference for comparison). In this study, GAPDH expression in tissues examined did not exhibit significant difference compared to the skin. (Grouping of SPX expression: No label, no difference *vs* the skin; ^(a)^ ≤ 5 fold higher than the skin; ^(b)^ between 5 to 10 fold higher than the skin; ^(c)^ ≥ 10 fold higher than the skin).

To shed light on tissue expression of SPX in mouse, RT-PCR was conducted in 28 tissues/organs and the results of real-time PCR revealed that transcript signals of SPX were consistently detected in all these samples (**Figure 3**). Using SPX expression in the skin as a reference for comparison, the tissues examined could be grouped into four categories, namely (i) the “low expression” group including the skin, skeletal muscle, lung and epididymis (with SPX signals similar to the skin), (ii) “medium-to-low expression” group including the fat, heart, thymus, esophagus, forestomach, small intestine, pancreas, kidney, prostate gland, uterus, and vagina (with SPX signals ≤ 5 fold higher than the skin), (iii) “medium-to-high expression” group including the glandular stomach, liver, adrenal gland, spleen, testes, and ovary (with SPX signals 5 to 10 fold higher than the skin), and (iv) “high expression” group including the colon and brain areas covering the olfactory bulb, cerebral cortex, hypothalamus, pituitary, cerebellum, and brain stem (with SPX signals ≥ 10 fold higher than the skin). Among the tissues examined, the brainstem was found to be the tissue with the highest level of SPX expression.

After the tissue expression profiling using RT-PCR, a more detailed histological study to unveil the cellular expression of SPX at protein level in selected tissues was carried out using IHS with antiserum raised against mammalian SPX. As a first step, the specificity of antiserum was tested by displacement studies with a SPX ELISA using the same antiserum. Based on our validation, the antiserum could recognize the amidated form of mouse SPX (i.e., the mature form of mouse SPX) and exhibit cross-reactivity with the amidated form of fish SPX but not the non-amidated counterparts (**Supplemental Figure 2A**) or kisspeptin and galanin (galanin-15 and -29) co-evolved from the same gene lineage (**Supplemental Figure 2B**). Similarly, cross-reactivity with unrelated peptides of comparable size, e.g., PACAP VIP, αMSH, NPY, secretin, and orexin A, was not detected (**Supplemental Figures 2C, D**). The specificity of the antiserum was further confirmed by IHS by replacement of SPX antiserum with a similar dilution of NRS (for the tissues selected for IHS) or with SPX antiserum pre-absorbed with a high dose of mouse SPX (for the liver and stomach only, see **Supplemental Figure 2E** for the results based on liver sections). In both cases, a total loss in SPX immunostaining signals could be observed.

**Figure 4:**
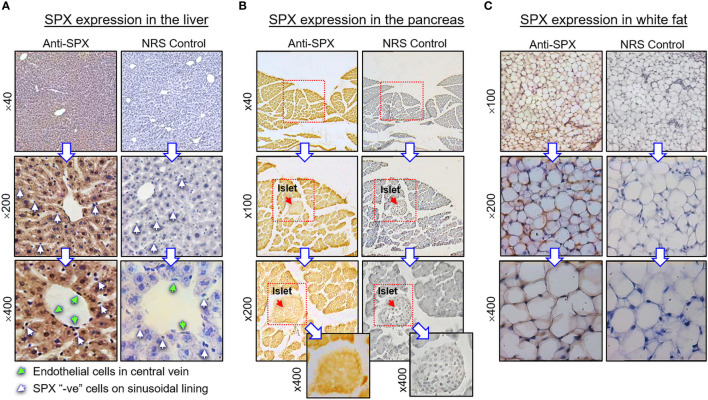
Histological distribution of SPX expression in the liver, pancreas and white fat. Immunoreactivity of SPX was detected by IHS in tissue sections (10 µm in thickness) prepared from **(A)** the liver, **(B)** pancreas, and **(C)** white fat of the mouse with SPX antiserum (anti-SPX, 1:600). In these cases, specific signals of SPX could be noted in the hepatocytes, endothelial cells in the central vein of hepatic lobules, endocrine cells in the islets of Langerhans, acinar cells of exocrine pancreas, and adipocytes forming the fat mass. Parallel staining with a similar dilution of normal rabbit serum (NRS) was used as the negative control and counterstaining with hematoxylin was routinely performed after signal development of SPX. The numbers shown on the side of individual panels (×40, ×100, ×200, and ×400) represent the magnification for the respective pictures.

**Figure 5:**
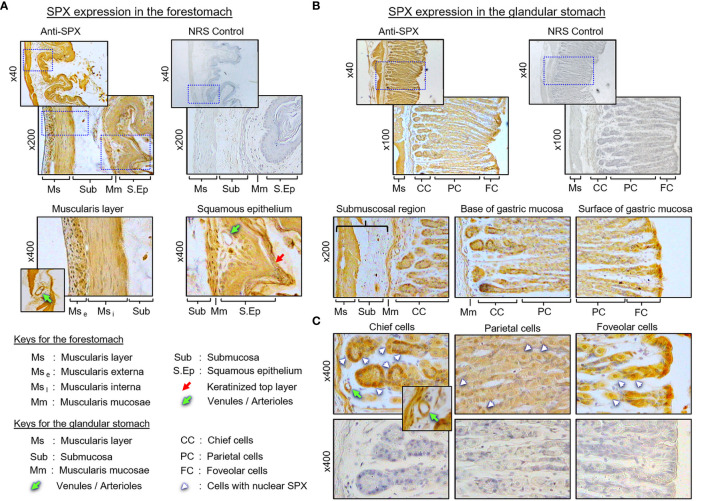
Histological distribution of SPX expression in the mouse stomach. **(A)** SPX expression in the forestomach. SPX immunoreactivity detected by anti-SPX was located in the muscularis layers and squamous epithelium, especially in muscularis externa, muscularis interna, muscularis mucosae, and squamous cell layers (including the keratinized layer on the top). **(B)** SPX expression in the glandular stomach. SPX signals were identified in the same muscularis layers as well as in the gastric glands of the mucosal layer. **(C)** SPX expression within the gastric mucosa. SPX immunostaining was observed in the foveolar cells, parietal cells and chief cells of the gastric glands. In the forestomach and glandular stomach, SPX signals were also noted in the endothelial cells and muscle layer of the venules/arterioles within/associated with the muscularis layers (insets). Parallel staining with NRS was used as negative control and the numbers shown on the side of individual panels (×40, ×100, ×200¸ and ×400) represent the magnification for the respective pictures.

**Figure 6:**
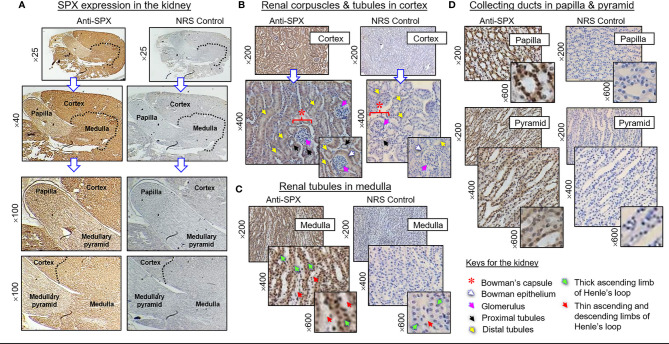
Histological distribution of SPX expression in the kidney. **(A)** SPX immunoreactivity detected by anti-SPX in different regions of the mouse kidney, including the papilla, medullary pyramid, inner medulla and outer cortex. **(B)** SPX expression in the outer cortex. SPX signals could be identified in Bowman epithelium (but not in glomerulus) of renal corpuscles as well as in the wall of both proximal and distal tubules. **(C)** SPX expression in the medulla. SPX signals were found in the wall of thick ascending limb but not in thin ascending/descending limbs of Henle’s loop. **(D)** SPX expression in the papilla and medullary pyramid. Specific signals of SPX could be noted in the wall of collecting ducts within the papilla and pyramid regions. Parallel staining with NRS was used as the negative control and the numbers shown on the side of individual panels (×25, ×40, ×100, ×200, ×400, and ×600) represent the level of magnification for the respective pictures.

**Figure 7:**
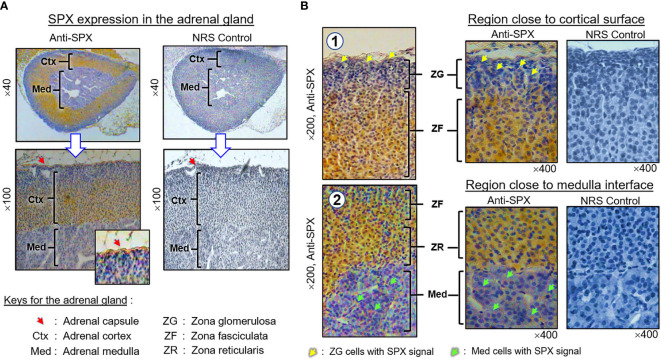
Histological distribution of SPX expression in the adrenal gland. **(A)** SPX immunoreactivity detected by anti-SPX in different regions of the mouse adrenal, including the adrenal capsule, outer cortex and inner medulla. **(B)** SPX expression in different cell layers of the outer cortex and within the inner medulla. In outer cortex, SPX signals were detected in adrenocortical cells of zona glomerulosa, zona fasciculata and zona reticularis, respectively. Faint signals of SPX immunostaining could also be noted in chromaffin cells of the inner medulla. In this study, parallel staining with NRS was used as the negative control and the numbers shown on the side/in the bottom (×40, ×100, ×200, and ×400) represent the magnification for the respective pictures.

**Figure 8:**
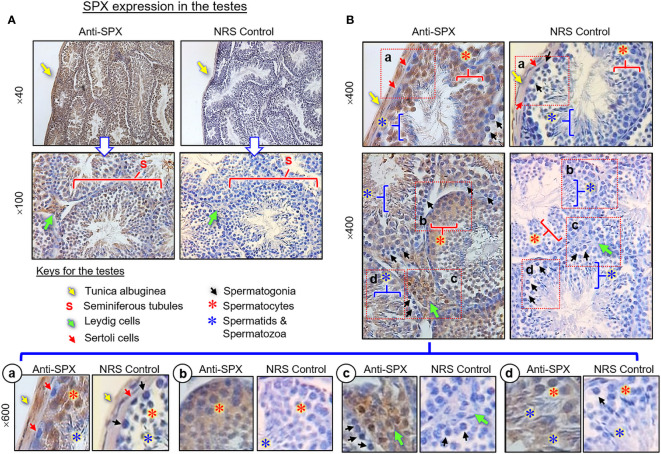
Histological distribution of SPX expression in the testes. **(A)** SPX immunoreactivity detected by anti-SPX in different regions of the mouse testes, including tunica albuginea, seminiferous tubules and interstitial areas with Leydig cells. **(B)** SPX expression in different cell types within seminiferous tubules. Except for Sertoli cells and spermatogonia, SPX signals could be noted in the spermatocytes, spermatid and mature spermatozoa **(**Subpanel a-d). In this study, parallel staining with NRS was used as the negative control and the numbers on the side (×40, ×100, ×400, and ×600) are the magnification of the respective pictures.

**Figure 9:**
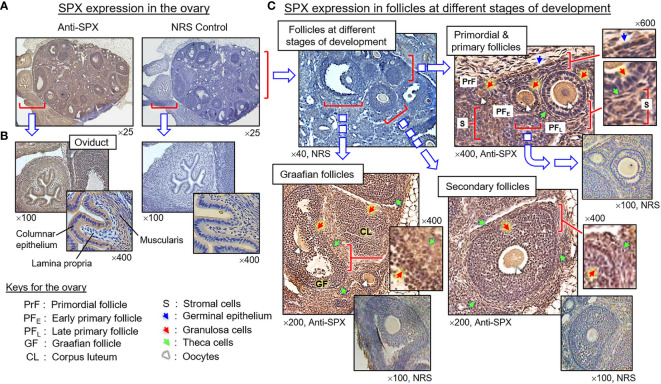
Histological distribution of SPX expression in the ovary. **(A)** SPX immunoreactivity detected by anti-SPX in the mouse ovary and oviduct. **(B)** SPX expression in the oviduct. SPX signals were located in columnar epithelium and muscularis layer but not in the lamina propria of the oviduct. **(C)** SPX expression in different cell types within the ovary. SPX immunostaining could be noted in the germinal epithelium, stromal cells, and various cell types (including the oocytes, granulosa cells and theca cells) within the follicles at different stages of development. In this study, parallel staining with NRS was used as the negative control and the numbers shown on the top/below individual panels (×40, ×100, ×200, ×400, and ×600) represent the magnification of the respective pictures.

**Figure 10:**
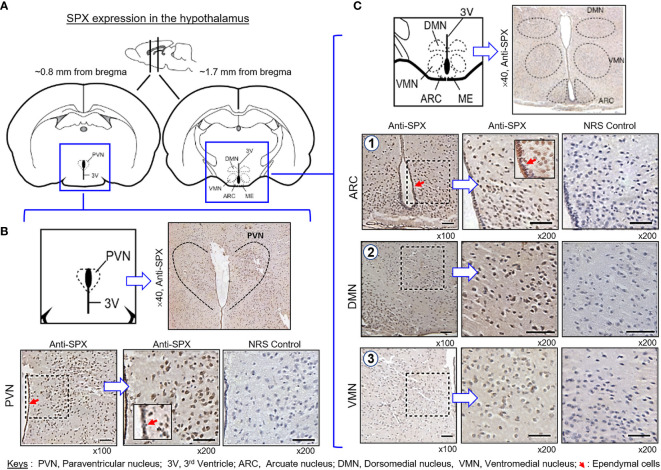
Histological distribution of SPX expression in the hypothalamus. **(A)** Brain sectioning to obtain the hypothalamus region for IHS with anti-SPX. Using the co-ordinates of stereotaxic atlas of mouse brain (with the bregma as a reference), cross-sections of the hypothalamus covering the areas of paraventricular nuclei (~0.8 mm from bregma) and dorsomedial, ventromedial and arcuate nuclei (~1.7 mm from bregma) were prepared and used for IHS study. **(B)** SPX expression in the anterior region of the hypothalamus. SPX immunoreactivity was detected in the glial cells forming the matrix of the hypothalamus, neuronal clusters forming the PVN, and ependymal cells in the boundary of the 3^rd^ ventricle. **(C)** SPX expression in the posterior region of the hypothalamus. SPX signals in glial cell matrix and ependymal cells surrounding the 3^rd^ ventricle could still be noted. SPX immunostaining was also found in the neuronal clusters forming the DMN, VMN and ARC nuclei. In this study, parallel staining with NRS was used as the negative control and the numbers on the side (×40, ×100, and ×200) represent the magnification of the respective pictures.

**Figure 11:**
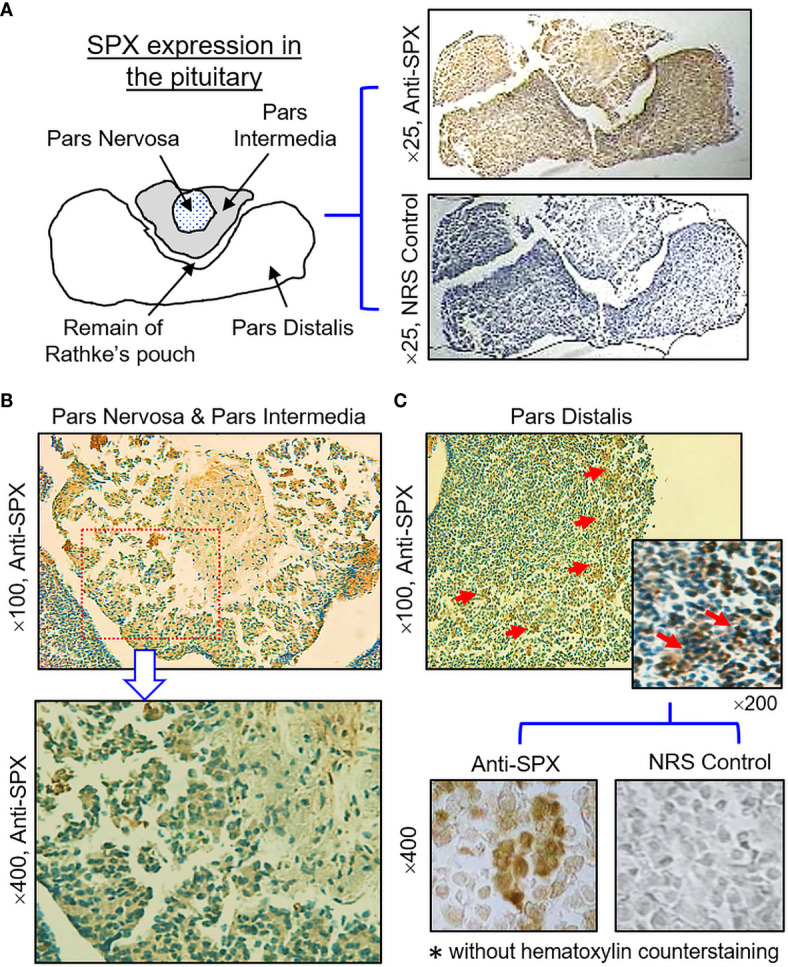
Histological distribution of SPX expression in the pituitary. **(A)** Immunoreactivity of SPX detected by anti-SPX in the mouse pituitary covering the areas of pars nervosa, pars intermedia and pars distalis. **(B)** SPX expression in the pars nervosa and pars intermedia. SPX signals were located in pituitary cells forming the matrix of pars nervosa and pars intermedia. **(C)** SPX expression in the pars distalis. SPX immunostaining was observed in cell clusters located in the outer edge of the pars distalis. In this study, parallel staining with NRS was used as the negative control and the numbers on the side (×25, ×100, ×200, and ×400) represent the magnification of the respective pictures.

**Figure 12:**
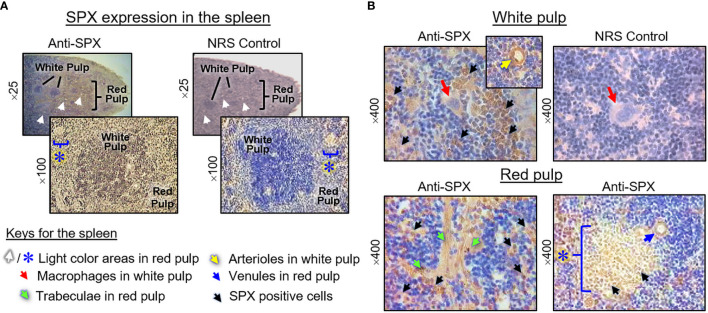
Histological distribution of SPX expression in the spleen. **(A)** SPX immunoreactivity detected by anti-SPX in different areas of the mouse spleen covering the white pulp and red pulp. **(B)** SPX expression in different cell types within the white pulp and red pulp. SPX signals could be located in the macrophages and distinct cell clusters within the white pulp (upper panels). In the red pulp, SPX immunostaining was detected in the trabeculae and cell clusters in their neighborhood as well as in the light color areas scattering among the red pulp. SPX signals were also observed in endothelial cells and muscle layer of the arterioles in the white pulp/venules in the red pulp. In this study, parallel staining with NRS was used as negative control and the numbers on the side (×25, ×100, ×200, and ×400) represent the magnification of the respective pictures.

**Figure 13:**
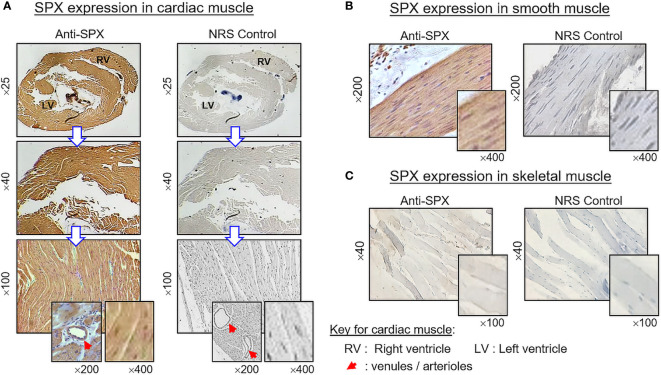
Histological distribution of SPX expression in different muscle types. SPX expression in **(A)** cardiac muscle, **(B)** smooth muscle, and **(C)** skeletal muscle as revealed by IHS with anti-SPX. SPX immunoreactivity could be detected in cardiac muscle forming different parts of the heart (e.g., left/right ventricles) and smooth muscle forming the muscularis layers of GI tract. However, it was not apparent in the myofibers of skeletal muscle. Similar to other tissues, SPX signals were found in the endothelial cells and muscle layer of arterioles/venules within the cardiac muscle (insets). In this study, parallel staining with NRS was used as negative control and the numbers on the side (×25, ×40, ×200, and ×400) represent the magnification of the respective pictures.

For cellular expression of SPX at the tissue level, the tissues related to endocrine functions/regulation or with documented functions for SPX were selected for our IHS study, and the results can be summarized as follows:

(I) SPX expression in the liver, pancreas, white fat, and stomach. In the liver, notable levels of SPX immunoreactivity were detected in the cytoplasm of hepatocytes forming the hepatic cords (**Figure 4A**). Similar signal was also located in the endothelial cells forming the inner lining of the central vein. Of note, some of the cells located on the surface of sinusoidal tracts (presumably the Kupffer cells) were found to be negative to SPX staining. The same is also true for the connective tissue forming the septa separating adjacent lobules of the liver. Similar to the liver, cytoplasmic expression of SPX could be noted in both the lobular structures of exocrine pancreas composed of acinar cells as well as in the endocrine cells within the islets of Langerhans (**Figure 4B**). Again, SPX signal was not found in connective tissue separating individual pancreatic lobules. For SPX signals in white adipose tissues, the pattern of SPX expression was pretty much identical for omental, visceral, inguinal, and peri-ovarian fat (**Figure 4C**, with omental fat as an example). In these cases, SPX staining was located in the cytoplasm of unilocular adipocytes but not in the central vacuole occupied by the lipid droplet. In the stomach, SPX signals were widely distributed in different regions of the forestomach (**Figure 5A** **)** and glandular stomach (**Figures 5B, C** **)**. In both cases, SPX immunoreactivity was noted in the muscle layers, including the muscularis interna and externa in the submucosal region and muscularis mucosae below the squamous epithelium in the forestomach or under the mucosal layer of glandular stomach (**Figures 5A, B**). In the forestomach, besides the muscle layers, SPX signals were also located in the squamous epithelium, especially in the keratinized layer close to the surface (**Figure 5A**). Similarly, SPX expression in glandular stomach could be found in the major cell types within the gastric mucosa, including the foveolar cells on the surface/in the neck of gastric pits, parietal cells forming the main body of gastric crypts, and chief cells located at the tips of individual gastric glands (**Figures 5B, C**). Although cytoplasmic staining of SPX was detected in different target cells in the stomach, nuclear expression of SPX could still be noted in distinct cell types, e.g., in foveolar cells, parietal cells, and chief cells. Furthermore, SPX signals were also observed in the wall of the venules/arterioles associated with the muscle layers (**Figure 5C** and its inset) but not in the connective tissues within the mucosal/submucosal regions.

(II) SPX expression in the kidney and adrenal gland. In the kidney, SPX immunoreactivity could be identified in different regions, including the papilla facing the renal pelvis, medullary pyramid forming the base of papilla, and inner medulla and outer cortex forming the main body of the kidney (**Figure 6A**). In the outer cortex, SPX signals were located in the wall of renal tubules (including both proximal and distal tubules), as well as in Bowman’s capsules (with signals expressed in Bowman epithelium but not the glomerulus) (**Figure 6B**). Within the medulla, SPX staining could be noted only in the wall of the thick ascending limb but not the thin ascending/descending limbs of the loop of Henle (**Figure 6C**). In the papilla and medullary pyramid, SPX signals were also detected in the wall of the collecting ducts leading to the ureter (**Figure 6D**). In the adrenal gland associated with the kidney, SPX immunoreactivity was expressed predominantly in the outer cortex with little signals in the inner medulla (**Figure 7A**). In the outer cortex, SPX signals could be found in the adrenal capsule (**Figure 7A**, inset) as well as in inner cell layers including the zona glomerulosa, zona fasciculata, and zona reticularis (**Figure 7B**). Unlike the notable signals of SPX with a uniform cytoplasmic distribution observed in adrenocortical cells, low levels of SPX immuno-staining were also detected in chromaffin cells within the inner medulla, and most of these signals were found to be located in the outer edge of SPX-positive cells (despite some of them still had faint signals in deeper part of the cytoplasm) (**Figure 7B**, lower panels).

(III) SPX expression in the testes and ovary. In the testes, SPX immunoreactivity was found in both the tunica albuginea and inner structures composed of seminiferous tubules and Leydig cells (**Figures 8A, B** **)**. Within the seminiferous tubules, SPX signals were not detected in Sertoli cells and spermatogonia attached to the basal lamina but could be recognized in spermatocytes within the basal cell layers and spermatids, spermatozoa, and residual bodies in adluminal region (**Figure 8B**, subpanels a–d). Similar to the testes, SPX immunostaining was widely distributed in different regions of the ovary and oviduct (**Figure 9A**). In the oviduct, SPX signals were located in the columnar epithelium and muscularis layer but not the lamina propria in between (**Figure 9B**). Within the ovary, immunoreactivity of SPX was found in the germinal epithelium on the surface, stromal cells forming the matrix of ovarian cortex, and developing follicles with increasing size, including the primordial follicles, primary follicles, secondary follicles, Graafian follicles, and newly formed corpus luteum (**Figure 9C**). At the follicle level, similar to the positive cells in the testes and oviduct, SPX signals were identified in the cytoplasm of granulosa cells, theca cells, and oocytes but not in the cavity of the antrum.

(IV) SPX expression in the hypothalamus and pituitary. Consistent with the results of RT-PCR, SPX immunoreactivity was detected in all the brain sections prepared along the long axis of the CNS in the mouse. To maintain our focus on the structures relevant to endocrine functions, only the data for SPX expression in the hypothalamo-pituitary axis are presented. Within the hypothalamus, SPX signals were detected in the glial cells forming the matrix of the midbrain area, ependymal cells lining the border of the 3rd ventricle, and neuronal clusters forming the distinct nuclei, including the paraventricular nuclei (PVN), dorsomedial nuclei (DMN), ventromedial nuclei (VMN), and arcuate nuclei (ARC) (**Figure 10**). At the pituitary level, SPX immunoreactivity could be noted in the pars nervosa extending from the hypothalamus, pars intermedia bordering the remain of Rathke’s pouch, and pars distalis forming the bulk of the anterior pituitary (**Figure 11A**). Interestingly, SPX signals were expressed ubiquitously in the cytoplasm of the cells within the pars nervosa and pars intermedia (**Figure 11B**) but a similar pattern of SPX expression was observed in individual cell clusters located mostly in the outer region of the pars distalis (**Figure 11C**).

(V) SPX expression in the spleen and different types of muscle. Within the spleen, immunoreactivity of SPX was found in both the white pulp and red pulp (**Figure 12A**). In the white pulp, SPX signals could be located in distinct cell clusters and macrophages (**Figure 12B**, upper panels) as well as in endothelial cells and muscle layer of the arterioles (**Figure 12B**, inset). Similarly, SPX immunostaining could also be noted in the trabeculae (with some of the cell clusters close by) and scattering light color areas within the red pulp, as well as in the wall of individual venules (**Figure 12B**, lower panels). Of note, except for the macrophages (with only cytoplasmic expression of SPX), most of the positive cells identified in the white/red pulp were found to have SPX signals expressed in the nucleus. Regarding SPX expression in the three major types of muscles, SPX immunoreactivity was ubiquitously expressed in the heart with cytoplasmic expression of SPX in individual cardiomyocytes. The same is true for SPX signals located in the wall of venules/arterioles within the cardiac muscle (**Figure 13A**). A similar pattern of SPX expression was also observed in smooth muscle prepared from different regions of the gut/stomach (**Figure 13B**, with smooth muscle from the colon as an example). Interestingly, immunostaining of SPX was not clearly discernable in skeletal muscle (**Figure 13C**, with gastrocnemius muscle as an example).

## Discussion

SPX is a 14 a.a. peptide first identified in human proteome using hidden Markov method (1), and its protein sequence is highly conserved among vertebrates (with only 1–2 a.a. substitutions from fish to mammals) (5, 6). Since the mature peptide of SPX is flanked by two dibasic protein processing sites (RR/KR and GRR) and with high levels of expression in major brain areas, it is commonly accepted that SPX is a neuropeptide with C-terminal α amidation (4, 5). Phylogenetic analysis coupled to comparative synteny reveal that SPX is co-evolved with galanin, despite a low level of sequence homology between the two peptides (4, 6). Of note, the biological actions of galanin are known to be mediated by three subtypes of galanin receptors, namely GalR1, GalR2, and GalR3, with differential coupling with cAMP/PKA, PLC/PKC, and Ca^2+^-dependent signaling cascades (44). Interestingly, functional expression of the three receptors in HEK293 cells also confirms that GalR2 and GalR3, but not GalR1, can serve as the cognate receptors for SPX (6). In mammals, galanin is a 29–30 a.a. gut-brain peptide with an α helix in the N-terminal followed by a β turn and a random coil in the C-terminal (45). Unlike the case of SPX, galanin has a strong preference for GalR1 and GalR2 but not GalR3 for receptor binding/activation (44). Based on the previous studies using truncated peptides of galanin, the ligand selectivity for the three receptors is highly dependent on the residues located in the α helix, including Gly^1^, Trp^2^, Asn^5^, Tyr^9^, and Gly^12^ for GalR1 binding and Trp^2^, Asn^5^, Gly^8^, and Tyr^9^ for GalR2 and GalR3 binding, and the C-terminal with a random coil appears to be not essential (40, 46). In parallel studies to map the binding sites at the receptor level by docking analysis and site-directed mutagenesis, TMD_3_, TMD_6_, and TMD_7_ together with ECL_2_ and ECL_3_ in GalR1 were shown to be the key structures forming the binding pocket for galanin docking (40, 47). However, only TMD_6_, TMD_7_, and ECL_3_ were required for the assembly of binding pocket in GalR2 (39, 46) and the same was also true for GalR3 but with additional involvement of TMD_3_ (48). At the molecular level, Gly^1^, Trp^2^, and Tyr^9^ in galanin have been reported to interact with Phe^115^ in TMD_3_, Phe^282^ in ECL_3_, and His^264^ in TMD_6_ of GalR1, respectively (47, 49). Furthermore, Gly^1^ of galanin is known to interact with Tyr^103^ in TMD_3_ of GalR3 (48), whereas the two consecutive His residues, His^252^ and His^253^, located in TMD_6_ of GalR2 can also interact with Trp^2^ in galanin (46). In contrast to the information for galanin docking, the structural interaction between SPX and GalR2/3 for receptor binding is largely unknown, and further investigations are clearly warranted.

Based on the reports in rodents and to a less extent in human, SPX is known to be widely expressed with pleiotropic functions in different tissues/organs (see *Introduction* for details). In our recent study with goldfish, SPX was shown to be a novel satiety factor with inhibitory effects on feeding behavior and food consumption (12) and the postprandial responses of SPX, both in the CNS and at the hepatic level, were mediated by PI3K/Akt and P_38_^MAPK^ cascades coupled to insulin secretion induced by glucose uptake (36). Although the mouse has been a major model for SPX studies, the mechanisms for feeding control by SPX is still unclear. As a first step to extend our study from fish to mammals, especially for the structural basis for SPX activation of GalR2/3, the solution structure of mouse SPX was established by NMR spectroscopies. Consistent with the prediction based on CD analysis, mouse SPX was shown to be a helical peptide composed of N-terminal random structure from Asn^1^ to Pro^4^ followed by an α helix motif covering Gln^5^ to Gln^14^ in the C-terminal. Apparently, its structural organization is opposite to that of galanin with the helical motif in the N-terminal. Using homology modeling of GalR2/3 and MD simulation of ligand docking, the α helix of SPX was confirmed to be a key structure for receptor binding similar to the case of galanin. Based on the models obtained, it can be inserted into the binding pocket formed by clustering of TMD_1–7_ of mouse GalR2/3, a typical feature of class A GPCR, and the molecular interactions are mediated by ionic bonding/hydrophobic contacts between Gln^5^, Leu^10^, Lys^11^, Gly^12^, Ala^13^, and Gln^14^ located in the helical region of SPX with specific residues within TMD_2 & 3_ and TMD_7_ for GalR2 and TMD_2_ and TMD_7_ for GalR3, respectively. Additional contacts can also be noted between the residues in the N- (Asn^1^ and Trp^2^) and C-terminal of SPX (Gly^12^, Alan^13^, and Gln^14^) with other residues in ECL_1 & 2_ for GalR2 and ECL_1, 2 & 3_ for GalR3, which is assumed to play a role in stabilizing the SPX : GalR2/3 complex. The involvement of the random coil in the N-terminal of SPX for receptor binding is quite different from the case of galanin, because its equivalent motif (i.e., the random coil in the C-terminal) is not involved in structural contact with galanin receptors, e.g., in human GalRs (39). By comparing the docking models for SPX binding in GalR2 and GalR3, the molecular contacts with SPX are largely distinct between the two receptors (especially for the contacts *via* Trp^2^, Gln^5^, Lys^11^, Gly^12^, Ala^13^, and Gln^14^ of SPX), which may play a key role in GalR subtype selectivity for SPX binding. However, overlapping contacts in GalR2 and GalR3 *via* highly conserved residues in TMD/ECL regions can still be noted, including (i) the H-bond between Asn^1^ in SPX and ECL_1_ (via Asp^88^ in GalR2 and Asp^86^ in GalR3) and (ii) the hydrophobic contacts *via* Leu^8^ in SPX with TMD_7_ (via Ile^274^ in GalR2 and Leu^274^ in GalR3) and Leu^10^ in SPX with ECL_2_ (via Val^173^ in GalR2 and Leu^171^ in GalR3) and TMD_2_ (via Ile^84^ in GalR2 and Ile^82^ in GalR3), respectively. Because the highly conserved contacts found in GalR2 and GalR3 for SPX docking are not overlapping with the TMD/ECL residues reported previously for galanin binding, it is tempting to speculate that these structural interactions not only can contribute to high affinity binding of SPX with GalR2 and GalR3 but also act as the key determinants for differentiating galanin vs SPX for ligand binding at the receptor level.

Although SPX signals are known to be widespread at tissue level, except for a single report in the rat (8) and a similar study restricted to endocrine/epithelial tissues in human (7), the previous studies on tissue distribution of SPX are mostly fragmentary and with a lack of details for SPX expression in different cell types in the target tissues. Given that (i) the mouse is a key model for SPX studies but with limited information for its tissue distribution, and (ii) there is a need of our project to identify target tissues with postprandial responses of SPX in the same model, a systematic study for tissue expression of SPX was conducted in the mouse. RT-PCR based on 28 tissues examined revealed that SPX was ubiquitously expressed in different tissues at transcript level. Apparently, SPX transcripts were highly expressed in the colon and different brain areas, and to a lower extent in the liver, glandular stomach, adrenal gland, spleen, and gonads. In the same study, moderate levels of SPX expression were detected in the heart, kidney, pancreas, white fat, esophagus, forestomach, small intestine, prostate gland, uterus, and vagina, and the signals were reduced to low levels in the skin, skeletal muscle, lung, and epididymis. To confirm the results based on RT-PCR, protein expression of SPX was also examined in 14 tissues with SPX functions/related to endocrine regulation by IHS using an antiserum validated to be highly specific for the mature peptide of mouse SPX. In agreement with transcript expression, SPX immunoreactivity could be located in hepatocytes within the liver, adipocytes in white fat, islet cells in endocrine pancreas and acinar cells in exocrine pancreas. In previous studies, SPX was shown to suppress bile acid synthesis at the hepatic level (11) and reduce fatty acid uptake in hepatocytes (16) and adipocytes of the mouse model (10), whereas SPX expression/secretion in hepatocytes has been recently demonstrated in fish model (e.g., goldfish) (36). In porcine islets, glucose treatment is known to alter SPX secretion (50), and SPX inhibition of insulin release/gene expression has been reported in rat islets/INS-E islet cells (15). These findings, together with ours, raise the possibility that SPX produced locally at tissue level may also contribute to the reported functions of SPX on lipid metabolism (16, 17) and glucose homeostasis (14) *via* autocrine/paracrine regulation of insulin output from the pancreas. Of note, our results on SPX expression in the pancreas, especially with SPX signals in both the islet cells and acinar cells, are highly comparable to the rat model (8). However, it is quite different from the case in human, in which the signal of SPX could be detected only in the islets of Langerhans but not in the acinar cells forming the matrix of pancreatic lobules (7). The cause of the discrepancy is unclear and may be due to species-specific variations. For SPX expression in acinar cells in rodents, the digestive function of acinar cells for enzyme release (e.g., amylase and lipase) into small intestine is well documented, but the biological actions of SPX in exocrine pancreas (e.g., enzyme synthesis/secretion) are still unknown and represent an unexplored area for further perusal.

In our study, SPX immunoreactivity was quite noticeable in cardiac muscle forming different parts of the heart and smooth muscle located in different regions of the GI tract (esophagus, stomach, small intestine, and colon). However, this is not the case for skeletal muscle, in which the signals of SPX were either weak (for SPX transcript) or not apparent (for SPX immunoreactivity). Although our results on SPX expression in smooth muscle and cardiac muscle are similar to the report in rat, SPX immunostaining was highly detectable in skeletal muscle of the rat model (8), indicating that species-specific variations can also exist in rodents. In the rat, SPX treatment is known to elevate arterial blood pressure with a concurrent drop in heart rate (24). Based on our findings, SPX signals not only could be found in the heart but also in the endothelial cells and muscle layer of arterioles/venules within the cardiac muscle, as well as in other tissues (e.g., stomach/spleen). Given the expression of SPX in key structures of the circulatory system, the possible involvement of SPX in cardiovascular functions in the mouse cannot be excluded. For SPX expression in smooth muscle of the GI tract, this is consistent with the report in rat for SPX-induced stomach contraction (1) and a recent study in mouse on SPX enhancement of bowel propulsion by GalR2 activation of smooth muscle contraction *via* Ca^2+^-dependent mechanisms (9). In the stomach, besides the signals in the muscle layers, SPX immunostaining was also observed in squamous epithelium of the forestomach (especially in the keratinized top layer) and different cell types forming the gastric glands within the glandular stomach (e.g., the mucus-producing foveolar cells, acid-secreting parietal cells, and enzyme-releasing chief cells). The findings of SPX signals in these “non-muscle” components of the stomach have prompted us to speculate that SPX may play a role in maintaining epithelial turnover in forestomach and/or contribute to digestive functions in glandular stomach of the mouse model.

Similar to the heart, notable levels of SPX immunostaining were identified in various regions of the mouse kidney, including the papilla, medullary pyramid, inner medulla, and outer cortex. In the cortex, SPX expression could be located in the wall of proximal and distal tubules as well as in the Bowman epithelium of renal corpuscles. Similar signals were also found in the thick ascending limb of Henle’s loop in the medulla region and in the collecting ducts within the papilla and medullary pyramid. Our findings corroborate with the previous report in the rat on SPX suppression of urine flow with a parallel drop in urinary Na^+^ excretion (24). However, similar IHS study in the rat kidney could only reveal SPX signals in the distal tubules but not in other renal structures (8), and the functional relevance for different patterns of SPX expression in the kidney of the two models is still unclear. For the endocrine organ associated with the kidney, namely the adrenal gland, predominant signals of SPX were detected in the adrenal cortex despite a low level of SPX immunostaining that could still be noted in the inner medulla. Protein expression of SPX in the adrenal gland has also been reported in the rat (8) and human (7). In agreement with our findings on SPX expression in the zona glomerulosa, zona fasciculata, and zona reticularis within the cortical layer, previous studies in rat adrenocortical cells have shown that SPX could suppress cell proliferation but elevate basal release of aldosterone and corticosterone (25). In the same report, SPX expression in the rat adrenal was found to be differentially regulated by signals from the hypothalamo-pituitary-adrenal axis, with stimulation by ACTH but inhibition by glucocorticoid. These findings, as a whole, suggest that SPX expression within the adrenal cortex may be a functional component mediating/contributing to the steroidogenic effect of ACTH, whereas the inhibition on SPX by glucocorticoid may indicate the presence of a negative feedback loop acting locally at the adrenal level. Although SPX signals could also be detected in the adrenal medulla, its effects on the functionality of chromaffin cells (e.g., on adrenalin synthesis/secretion) are still unknown.

Consistent with the functional role of SPX as a neuromodulator/neurotransmitter within the CNS (4), high levels of SPX expression (especially for transcript signals) could be located in different brain areas of the mouse. These results are comparable to similar reports in fish models (e.g., goldfish) (12, 18) and lend support to the ideas for the central actions of SPX in anorexia nervosa/psychiatric disorder (33, 34), anxiety/depression (22, 23), and pain perception (20, 21). In our study, the brainstem was found to be the brain area with the highest level of SPX signal, which is also a key structure for coordination and processing of nociceptive signals between the cerebrum/cerebellum and spinal cord (51). In the brain, similar to the transcript signals, SPX immunoreactivity could be noted in the hypothalamo-pituitary axis. In the hypothalamus, protein signals of SPX were found in the glial cells, ependymal cells as well as in neuronal structures forming the PVN, DMN, VMN, and ARC nuclei, which are the major brain areas with neuroendocrine functions. At the pituitary level, similar signals were expressed in pituitary cells forming the matrix of pars nervosa and pars intermedia, as well as in cell clusters scattering in the outer edge of pars distalis. Despite the lack of information for SPX actions in the hypothalamo-pituitary axis in mammals, previous studies in fish models have reported that hypothalamic expression of SPX could be altered by food intake (e.g., goldfish and ya fish) (12, 52) or prolonged period of fasting (e.g., sole and spotted scat) (53, 54). In goldfish, consistent with the inhibitory effects of SPX on feeding behavior and food consumption, ICV injection of SPX was shown to up-regulate POMC with concurrent reduction of NPY and AgRP expression in the hypothalamus (12), and the postprandial signal of insulin was also effective in stimulating central expression of SPX *via* activation of PI3K/Akt and P_38_^MAPK^ pathways (36), implying that hypothalamic expression of SPX is an integral component of the feeding circuitry within the brain. In the same animal model, SPX could also suppress LH secretion in the pituitary, and hypothalamic expression of SPX was found to be sensitive to estrogen feedback (18). These findings have been confirmed by recent studies in tilapia (for LH inhibition) (19) and spotted scat (for estrogen effect) (53), implying that SPX expression in the hypothalamo-pituitary axis is involved in reproductive functions in fish species. Given that SPX signals could be identified in different regions of the mouse pituitary, especially in pars nervosa and intermedia, the functional role of SPX in regulating pituitary hormones, e.g., neural lobe hormones/gene products of POMC, for sure can be an interesting direction for future studies in mouse model.

In agreement with the reproductive function proposed for SPX, notable levels of SPX signals (both transcripts and immunoreactivity) were found to be expressed in the gonads of the mouse model. In the testes, except for a lack of signal in Sertoli cells and spermatogonia, SPX immunostaining was located in Leydig cells within the interstitial areas and spermatocytes, spermatids and mature spermatozoa in seminiferous tubules. In the ovary, similar signals were detected in germinal epithelium, stromal cells, and follicles at different stages of development. At the follicular level, SPX signals could be identified in the oocyte, as well as in granulosa and theca cell layers. The patterns of gonadal expression of SPX observed are comparable and yet distinct from the study in rat model. In contrast to the mouse, SPX is not detectable in the oocyte within the ovary, and Leydig cells and Sertoli cells are the major cell types with SPX expression in the testes of the rat (8). Given that (i) Leydig cells in the male/follicular cells in the female are key structures for production of sex steroids, and (ii) SPX signal was expressed in the gametes of both sexes at different stages of development/maturation, the possible involvement of SPX in steroidogenesis and/or gametogenesis cannot be excluded. In the mouse, SPX immunoreactivity was also detected in the columnar epithelium and muscularis layer of the oviduct. Judging from the previous study on SPX induction of smooth muscle contraction (e.g., in the gut) (9), it is likely that SPX may play a role in embryo transport to the uterus after fertilization. In our study, the spleen, a major organ in the lymphatic system, was found to be another structure with SPX expression at the transcript and protein levels. Similar to the findings in the rat (8), SPX immunostaining could be noted in cell clusters and macrophages in the white pulp and different areas (e.g., in the trabeculae and light color areas) of the red pulp. However, the functional role of SPX in the spleen, especially related to immune responses, is still unknown.

In summary, we have established the NMR solution structure of mouse SPX and reveal that it is a helical peptide with an N-terminal random coil from Asn^1^ to Pro^4^ followed by an α-helix from Gln^5^ to Gln^14^ in the C-terminus. Using homologous modeling and MD simulation, the molecular docking of SPX with mouse GalR2/3 was deduced with mapping of the “contact sites” between the ligand and the respective receptors. Using RT-PCR and IHS, the tissue distribution profile and histological expression of SPX at the tissue level have also been characterized in the mouse. Our studies not only confirm the role of GalR2/3 as the cognate receptors for SPX with a structural approach, but also provide useful data for the molecular interactions between SPX and GalR2/3 during receptor binding. This information will form the basis of our on-going study with site-directed mutagenesis of SPX and GalR2/3, which will have a strong implication on the design of SPX-based pharmacological agents for therapeutic use (42). As mentioned in the *Introduction*, the tissue expression of SPX was conducted to serve as the initial study to allow us to identify the potential targets with postprandial responses of SPX expression (for the results of these studies, please refer to part II of this three-paper series). Although our data on tissue distribution of SPX are descriptive in nature, our results based on IHS study do provide the anatomical basis for possible functions of SPX in a couple of tissues, e.g., for reproductive function at the gonadal level, immune responses in the spleen, digestive function in the stomach, and neuroendocrine regulation in the adrenal gland and hypothalamo-pituitary axis. Furthermore, besides the cytoplasmic expression of SPX found in most of the tissues examined, SPX immunoreactivity was also detected in the nuclei of different cell types in the gastric gland of the stomach, as well as in distinct cell clusters in the white/red pulp of the spleen. The nuclear function of SPX is totally unknown and may represent a new area of research for SPX in the mouse model.

## Data Availability Statement

The original contributions presented in the study are included in the article/**Supplementary Material**. Further inquiries can be directed to the corresponding author.

## Ethics Statement

The animal study was reviewed and approved by the committee on the Use of Live Animal in Teaching and Research, The University of Hong Kong, Hong Kong.

## Author Contributions

AW was the PI and grant holder. AW, MW, and MH were responsible for project planning and data analysis. KS and TH were in charge of NMR spectroscopy and docking models of SPX binding, respectively. MW, MH, and WK were responsible for RT-PCR and IHS staining for SPX expression at tissue level. Manuscript preparation was done by AW and Z-XB. All authors contributed to the article and approved the submitted version.

## Funding

The project was supported by HMRF grant (13142591), Food & Health Bureau (HKSAR), GRF Grants (17105819, 17113918, and 17117716), and Research Grant Council (Hong Kong).

## Conflict of Interest

The authors declare that the research was conducted in the absence of any commercial or financial relationships that could be construed as a potential conflict of interest.

## Supplementary Material

Supplementary Figure 1 Ramachandran analysis for quality control of structural data for homology modeling of mouse GalR2 and GalR3. The Phi-Psi plots of the respective data for **(A)** GalR2 and **(B)** GalR3 reveal that all the data are within the most favored region (green dots within the areas covered by blue line) and additional allowed region (yellow dots within the areas covered by orange line) but not in the generously allowed region/disallowed region, indicating that the receptor modeling are of good quality.

Supplementary Figure 2 Validation of antiserum specificity for detection of SPX. The specificity of the antiserum for mouse SPX was tested in an ELISA system using biotinylated mouse SPX as the tracer for displacement studies with **(A)** amidated and non-amidated forms of mouse SPX (m.SPX) and fish SPX (f.SPX), **(B)** related peptides evolved from the same gene lineage, including kisspeptin, galanin 15 and galanin 29, **(C, D)** unrelated peptide with similar size, including PACAP, VIP, secretin, NPY, α MSH and orexin A. In these studies, except for m.SPX and f.SPX (with ED_50_ of 45.3 pg/ml and 32.9 ng/ml, respectively), the other peptides were not effective in displacing the binding of the SPX tracer.

## Abbreviations

NPY, neuropeptide Y; AgRP, agouti-related peptide; CCK, cholecystokinin; CART, cocaine- and amphetamine-regulated transcript; POMC, pro-opiomelanocortin; VIP, vasoactive intestinal peptide; PACAP, pituitary adenylate cyclase-activating polypeptide; CD, circular dichroism; NMR, nuclear magnetic resonance; TOCSY, total correlation spectroscopy; DQF-COSY, double-quantum filtered correlation spectroscopy; NOESY, nuclear Overhauser effect (NOE) spectroscopy; MD simulation, molecular dynamics (MD) simulation; IHS, immunohistochemical staining; ECL/ICL, extra/intracellular loop; TMD, transmembrane domain; Sub, submucosa; Ms_e/i_, muscularis externa/interna; Mm, muscularis mucosae; S.Ep, squamous epithelium; CC, chief cells; PC, parietal cells; FC, foveolar cells; PrF, primordial follicle; PF_E/L_, early/late primary follicle; GF, Graafian follicle; CL, corpus luteum; 3V, 3^rd^ ventricle; PVN, paraventricular nucleus; DMN, dorsomedial nucleus; VMN, ventromedial nucleus; ARC, arcuate nucleus; Ctx, adrenal cortex; Med, adrenal medulla; ZG, zona glomerulosa; ZF, zona fasciculate; ZR, zona reticularis.
