# Supplementary material for: Mouse Spexin: (I) NMR Solution Structure, Docking Models for Receptor Binding, and Histological Expression at Tissue Level
Source: Front Endocrinol (Lausanne). 2021 Jul 2;12:681646. doi: 10.3389/fendo.2021.681646 (PMC8285161; doi:10.3389/fendo.2021.681646)
Supplement: Supplementary Figure 1 — Ramachandran analysis for quality control of structural data for homology modeling of mouse GalR2 and GalR3. The Phi-Psi plots of the respective data for (A) GalR2 and (B) GalR3 reveal that all the data are within the most favored region (green dots within the areas covered by blue line) and additional allowed region (yellow dots within the areas covered by orange line) but not in the generously allowed region/disallowed region, indicating that the receptor modeling are of good quality. [file DataSheet_1.pdf]

Supplemental Table 1

Structure calculation statistics

	Mouse SPX
Nonredundant upper-limit distance constraints	159
Short range i-j = 1	106
Medium range 1 < i-j <5	49
Long range i-j > = 5	4
Residual NOE target function (Å^2)	0.03
Residual NOE distance limit violations	
≥0.2 Å	0
Maxium (Å)	0.01
Average violation (Å)	0.0006
RMSD from the mean coordinates (Å)	Residues 5–13
Backbone atoms	0.02 ± 0.01
All heavy atoms	0.34 ± 0.09
Ramachandran statistics for Φ and Ψ	
Residues in most favored region (%)	100.0%
Residues in additional allowed region (%)	0.0%
Residues in generously allowed region (%)	0.0%
Residues in disallowed region (%)	0.0%

(NOE, Nuclear Overhauser effect; RMSD, Root mean square deviation)
