# Supplementary material for: Mouse Spexin: (I) NMR Solution Structure, Docking Models for Receptor Binding, and Histological Expression at Tissue Level
Source: Front Endocrinol (Lausanne). 2021 Jul 2;12:681646. doi: 10.3389/fendo.2021.681646 (PMC8285161; doi:10.3389/fendo.2021.681646)
Supplement: Supplementary Figure 2 — Validation of antiserum specificity for detection of SPX. The specificity of the antiserum for mouse SPX was tested in an ELISA system using biotinylated mouse SPX as the tracer for displacement studies with (A) amidated and non-amidated forms of mouse SPX (m.SPX) and fish SPX (f.SPX), (B) related peptides evolved from the same gene lineage, including kisspeptin, galanin 15 and galanin 29, (C, D) unrelated peptide with similar size, including PACAP, VIP, secretin, NPY, α MSH and orexin A. In these studies, except for m.SPX and f.SPX (with ED50 of 45.3 pg/ml and 32.9 ng/ml, respectively), the other peptides were not effective in displacing the binding of the SPX tracer. [file DataSheet_2.pdf]

Supplemental Fig.1

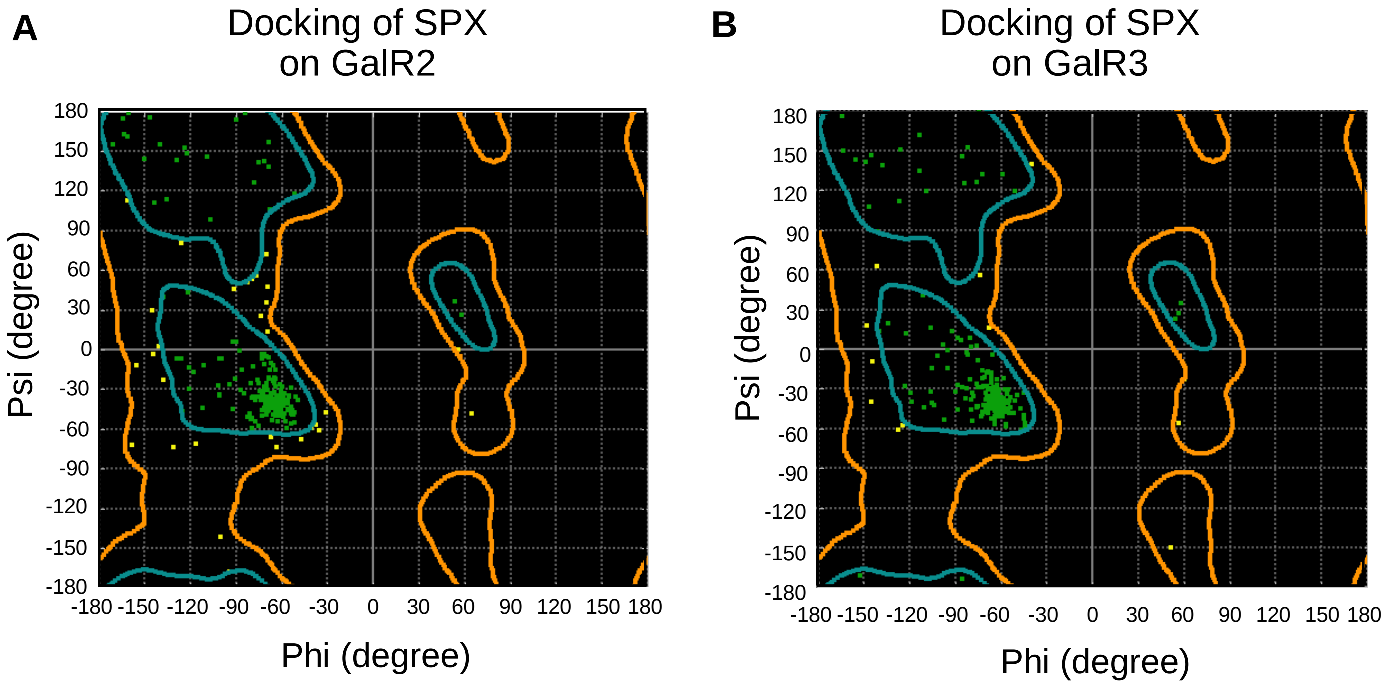

Ramachandran analysis for quality control of structural data for homology modelling of mouse GalR2 and GalR3. The Phi-Psi plots of the respective data for (A) GalR2 and (B) GalR3 reveal that all the data are within the most favored region (green dots within the areas covered by blue line) and additional allowed region (yellow dots within the areas covered by orange line) but not in the generously allowed/disallowed region, indicating that the results of our receptor modelling are of good quality.
