# Supplementary material for: Mouse Spexin: (I) NMR Solution Structure, Docking Models for Receptor Binding, and Histological Expression at Tissue Level
Source: Front Endocrinol (Lausanne). 2021 Jul 2;12:681646. doi: 10.3389/fendo.2021.681646 (PMC8285161; doi:10.3389/fendo.2021.681646)
Supplement: Supplementary file 3 [file DataSheet_3.pdf]

Supplemental Fig.2

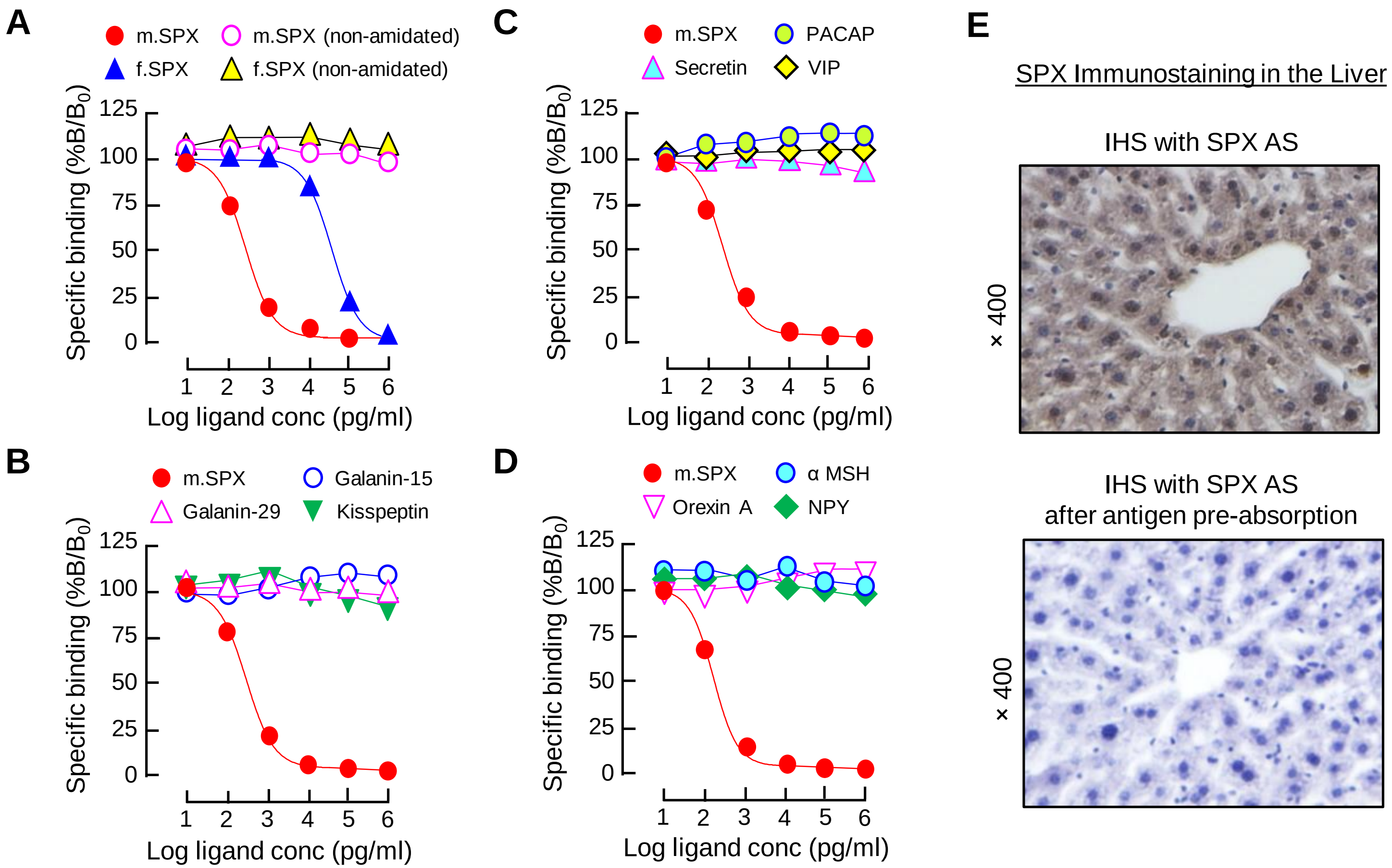

Validation of antiserum specificity for detection of SPX. The target specificity of the antiserum for mouse SPX was tested in an ELISA system using biotinylated mouse SPX as the tracer for displacement studies with (A) amidated and non-amidated forms of the mouse (m.SPX) and fish SPX (f.SPX), (B) related peptides co-evolved from the same gene lineage, including kisspeptin, galanin-15 and galanin-29, (C & D) unrelated peptide with similar size, including PACAP, VIP, NPY, α MSH, secretin and orexin A. In these studies, except for m.SPX and f.SPX (with ED_{50} of 45.3 pg/ml & 32.9 ng/ml, respectively), the other peptides were not effective in displacing the binding of SPX tracer. In parallel study, antiserum specificity was also confirmed by antigen pre-absorption followed by IHS staining in liver sections (E). Liver sections prepared from the mouse were subjected to immunostaining with SPX antiserum (SPX AS, 1:600) with/without prior incubation with mouse SPX (20 mM, 12 hr at 4 ^{o}C) according to the standard procedures. In this case, pre-absorption with SPX was found to abolish the immunostaining signal of SPX at the tissue level.
